# The Elucidation of the Molecular Mechanism of the Extrusion Process

**DOI:** 10.3390/ma14154278

**Published:** 2021-07-30

**Authors:** Joanna Doskocz, Paulina Dałek, Magdalena Przybyło, Barbara Trzebicka, Aleksander Foryś, Anastasiia Kobyliukh, Aleš Iglič, Marek Langner

**Affiliations:** 1 Department of Biomedical Engineering, Faculty of Fundamental Problems of Technology Wrocław, University of Science and Technology, pl. Grunwaldzki 13, 50-377 Wrocław, Poland; paulina.dalek@pwr.edu.pl (P.D.); magdalena.przybylo@pwr.edu.pl (M.P.); marek.langner@pwr.edu.pl (M.L.); 2 Lipid Systems sp. z o.o., ul. Krzemieniecka 48C, 54-613 Wrocław, Poland; 3 Centre of Polymers and Carbon Materials, Polish Academy of Sciences, ul. M. Curie-Sklodowskiej, 41-819 Zabrze, Poland; btrzebicka@cmpw-pan.edu.pl (B.T.); aforys@cmpw-pan.edu.pl (A.F.); akobyliukh@cmpw-pan.edu.pl (A.K.); 4 Laboratory of Physics, Department of Fundamentals of Electrical Engineering, Mathematics and Physics, Faculty of Electrical Engineering, University of Ljubljana, Tržaška 25, SI-1000 Ljubljana, Slovenia; ales.iglic@fe.uni-lj.si; 5 Laboratory of Clinical Biophysics, Department of Orthopedics, Faculty of Medicine, University of Ljubljana, Zaloška 9, SI-1000 Ljubljana, Slovenia

**Keywords:** liposomes, extrusion, lipid bilayer, membrane mechanics, bending rigidity

## Abstract

Extrusion is a popular method for producing homogenous population of unilamellar liposomes. The technique relies on forcing a lipid suspension through cylindrical pores in a polycarbonate membrane. The quantification of the extrusion and/or recalibration processes make possible the acquisition of experimental data, which can be correlated with the mechanical properties of the lipid bilayer. In this work, the force needed for the extrusion process was correlated with the mechanical properties of a lipid bilayer derived from other experiments. Measurements were performed using a home-made dedicated device capable of maintaining a stable volumetric flux of a liposome suspension through well-defined pores and to continuously measure the extrusion force. Based on the obtained results, the correlation between the lipid bilayer bending rigidity and extrusion force was derived. Specifically, it was found that the bending rigidity of liposomes formed from well-defined lipid mixtures agrees with data obtained by others using flicker-noise spectroscopy or micromanipulation. The other issue addressed in the presented studies was the identification of molecular mechanisms leading to the formation of unilamellar vesicles in the extrusion process. Finally, it was demonstrated that during the extrusion, lipids are not exchanged between vesicles, i.e., vesicles can divide but no membrane fusion or lipid exchange between bilayers was detected.

## 1. Introduction

The mechanics of the lipid bilayer are one of the central issues of contemporary biology and a critical element for the development of liposome-based drug delivery systems [1,2] and other supramolecular nanodevices such as biosensors for environmental monitoring and medical diagnostics [3,4,5,6,7,8,9] or cargo capsules for cosmetics, nutraceuticals, and food additives [10,11,12,13,14]. Polar lipids are convenient materials for the construction of drug delivery systems in the form of liposomes (a closed spherical lipid bilayer) [15]. In order to be used as effective nanodevices, liposomes, as well as their production processes, need to be parametrized so the resulting pharmacological or medical products can pass the scrutiny of rigorous formal approval processes [16]. Whereas some parameters, such as liposome size distribution or encapsulation efficiency, can be reliably quantitated, others, such as mechanical properties, are rarely measured. However, based on theoretical and experimental studies, it was demonstrated that elasticity affects the rate of nanoparticles internalization by cells and accumulation in the tissues [17,18,19,20,21,22]. The mechanics of a lipid bilayer will also affect the formation protocols of liposomes [10,23,24,25,26,27,28,29]. The scientific and technological importance of liposome mechanics is unmatched by the development of reliable and quantitative experimental methods.

Bending rigidity (κ) and bilayer tensile strength $\left(\gamma \right)$ are parameters that quantitate the mechanical properties of the lipid bilayer, which is the building structure of liposomes. The bending rigidity determines the amount of energy that must be applied to bend a lipid bilayer [30]. It depends on the lipid bilayer intrinsic properties, as well as environmental conditions [30,31,32,33,34,35,36,37]. Currently available techniques, used to evaluate lipid bilayer rigidity, can be classified into the following qualitatively different categories [38]:

i. Fluctuation spectroscopy, which is based on shape deformations of giant unilamellar vesicles (GUVs) [32,39,40,41,42],
ii. Methods based on membrane deformation induced by an external force, where the lipid bilayer elastic properties are calculated using the relation between the force and the extent of membrane deformation (e.g., micropipette aspiration, optical tweezer, electrodeformation, and Atomic Force Microscopy (AFM) manipulations) [10,31,43,44,45,46,47,48],
iii. Scattering techniques [49,50,51,52], where structural parameters of supported lipid bilayers are correlated with mechanical properties,
iv. Molecular dynamics simulation [53,54], where mechanical properties are derived from the simulation of small lipid bilayer fragments,
v. Estimation of the mechanical properties using theoretical methods of soft matter physics [55,56],
vi. Monte Carlo simulations [57].

The technological application of either of those methods for the evaluation of liposomes is impractical due to the inconvenient experimental models, complex experimental infrastructure, or assumption-limited theoretical models used. Fluctuation spectroscopy and the aspiration technique use a difficult-to-validate experimental model, i.e., liposomes with a diameter greater than 1 µm (GUV). In addition, methods based on GUVs are technically demanding and cannot be easily adopted to complex lipid mixtures, due to the potentially heterogenous vesicle population. Critically, the measured values cannot be easily translated to the submicron scale. Molecular dynamic simulations, theoretical modeling, and Monte Carlo simulations cannot be used alone; hence, they require experimental validation of the derived quantitative values. The method based on the scattering technique uses stacks of supported lipid bilayers as an experimental model system, so direct liposome measurement is not possible. In addition, the technique requires a complex theoretical model, the assumptions of which are difficult to verify [58]. Consequently, the effective development of liposome-based drug delivery systems is easy to implement, effective, reliable, and capable of providing quantitative measures of the final version of liposomes intended for pharmacological applications.

The lipid bilayer tensile strength can be measured by applying a pressure difference across the lipid bilayer [59]. The pressure difference may result from osmotic imbalance [60] or a hydrostatic pressure difference may be generated between aqueous compartments of GUV [59,61,62].

**Figure 1:**
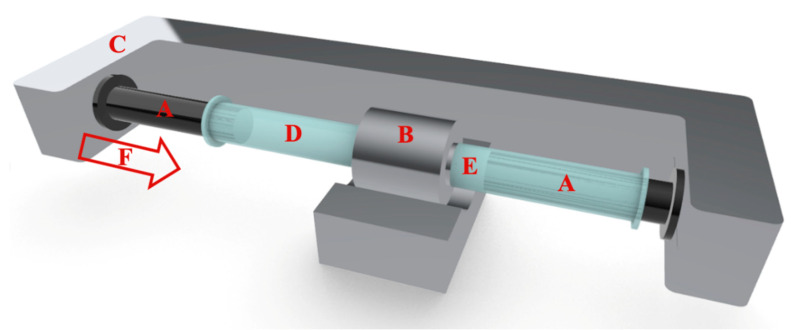
Schematic diagram of an automated extrusion device. The system consists of two Hamilton Syringes (**A**), a holder for the filter (**B**), and an arm powered by a stepper motor (**C**). Liposome suspension before (**D**) and after extrusion (**E**) and Extrusion force (**F**) are indicated in the drawing.

Recently, we have introduced a measuring technique, easy to implement into industrial settings, for the determination of the mechanical properties of a lipid bilayer [63]. The method is based on the measurement of the force needed to support the predetermined volumetric flow through the nanopores of the extrusion membrane. Extrusion is one of the most common methods for producing a homogenous unilamellar liposome suspension (LUV) [64]. Typically, a multilamellar liposome suspension (MLV) is passed through a membrane filter to obtain liposomes with a mean diameter correlated with the diameter of pores in the filter [65]. The extrusion is carried out manually or using a high-pressure difference to force the liposome suspension through the filter [65,66,67]. In our work, an automated extruder was used (Figure 1). The device consists of a step-motor and strain gauges, so the piston is capable of maintaining a constant volumetric flux of liposome suspension through the membrane pores when continuously monitoring the required temporal extrusion force. The mechanism of unilamellar vesicles creation using the extrusion technique is not well understood. There is still a lack of a consistent model explaining how large, multilamellar liposomes with diameters orders of magnitude larger than pores break up into smaller, unilamellar vesicles during the extrusion process [68]. It has been observed that the average size of liposomes obtained by the extrusion method is, in some cases, greater than the diameter of pores in the filter, indicating the liposome shape alteration when inside the pore (if the filter with a pore diameter smaller than 200 nm is used) [68,69]. Numerous experimental and theoretical studies aiming at the correlation between properties of the lipid bilayer and parameters of the extrusion process have been reported, but no mechanistic description of the process has been offered [68,70,71,72,73,74]. In the present work, liposome formation and/or recalibration processes (preformed LUVs are forced through pores of significantly smaller sizes [75]) were used to evaluate the mechanical properties of a lipid bilayer, i.e., membrane bending rigidity and tensile strength. The liposome formation process involves the transformation of heterogenous multilamellar vesicle suspensions into a homogenous suspension of unilamellar vesicles by forcing them through the filter with a specific pore size. The “recalibration” is understood here as a process when a homogenous unilamellar liposome suspension is forced through the filter with a pore size smaller than the average size of liposomes in the suspension. Depending on the properties of a lipid bilayer, the outcome of the procedure may vary. Finally, the molecular mechanism of the extrusion process is proposed.

## 2. Materials and Methods

### 2.1. Materials

Lipids: POPC (1-palmitoyl-2-oleoyl-sn-glycero-3-phosphocholine), cholesterol, and fluorescent probes, namely, NBD (1-acyl-2-(6-[(7-nitro-2-1,3-benzoxadiazol-4-yl)amino]hexanoyl)-sn-glycero-3-phosphocholine) and Rh-B (18:1 Liss Rhod PE 1,2-dioleoyl-sn-glycero-3-phosphoethanolamine-N-(lissamine rhodamine B sulfonyl) ammonium salt), were purchased from Avanti Polar Lipids (Alabaster, AL, USA). Chloroform and sodium chloride were obtained from VWR sp. z o.o. (Gdańsk, Poland). Ferric chloride hexahydrate and ammonium thiocyanate were purchased from Chempur (Łódź, Poland). Polycarbonate membranes (Nuclepore Corp., Pleasanton, CA, USA) and 2.5 mL syringes (Hamilton Bonaduz, Bonaduz, Switzerland) were used for the extrusion process, and 18 MΩ of deionized water was used in all experiments (PolWater, Kraków, Poland).

### 2.2. Quantitative Method for the Evaluation of the Liposome Extrusion

Multilamellar liposomes (MLVs) were prepared by the dry film method [76]. First, lipids were dissolved in chloroform (with a fluorescent probe in the amount of 0.5 mol%, if applicable). Then, the organic solvent was evaporated under a stream of argon (AirProducts sp. z o.o., Warszawa, Poland) and the remaining chloroform was removed under vacuum (Vacuum pump Rocker 410, Rocker, Kaohsiung City, Taiwan). Next, the dry lipid film was hydrated with water or a saline solution followed by vortexing (Vortex MX-S Chemland, Stargard, Poland) and hydration overnight. The obtained MLV suspension was extruded using an automated mechanical extruder (Lipid Systems Ltd., Wrocław, Poland) to produce a population of the unilamellar vesicles (Figure 1) [77]. As described elsewhere, the extrusion process was performed using the device capable of maintaining a constant value of the volumetric flow of the liposome suspension through the filter and continuously monitoring the extrusion force [63]. The volumetric flux (*J_V_*) across the polycarbonate filter was calculated according to the formula:

(1) $$J_{V}=\frac{R_{s}^{2}\cdot v_{p}}{R_{F}^{2}}$$

where $v_{p}$ is the velocity of the piston in the syringe, which was set before each measurement, whereas *R_S_* and *R_F_* stand for the syringe and filter radii, respectively.

In previous work [63], it has been shown that the extrusion force depends on measurement conditions such as the volumetric flux of the liposome suspension through pores, temperature, pore diameter, and concentration of lipids in the suspension. In order to determine the effect of lipid composition of the lipid bilayer or test the proposed mechanisms of the liposome formation process, the experimental conditions had been fixed, specifically, the lipid concentration was set to 10 mg/mL, the volumetric flux was maintained at 0.48 (mL∙min^−1^∙cm^−2^), and pores in the filter had diameters equal to 0.1 µm if not stated otherwise.

### 2.3. Liposome Suspension Characterization

Liposomes size distributions and polydispersity indexes (PDI) were measured with the dynamic light scattering method (DLS) (Zetasizer Nano ZS, Malvern, UK) after samples were diluted 50 times with the deionized water or buffer (Chempur, Łódź, Poland). All solutions before use were filtered through the cellulose membrane with 0.2 μm pores (VWR, Gdańsk, Poland).

The lipid concentration in the liposome suspension, before and after the extrusion process, was determined by the Stewart method [78]. Specifically, 40 µL of liposome suspension was added to 2 mL of chloroform, 2 mL of 0.4 M of ammonium ferrothiocyanate, and 0.1 M of ferric chloride hexahydrate solution. Then, the samples were vortexed and set aside for 15 min. The lipid concentration was calculated based on the determined value of absorbance (SPECTROstar Nano, BMG LABTECH, Ortenberg, Germany) and the calibration curve. The liposomes fluorescence was measured using a Fluoromax-4 Spectrofluorometer (Horiba, Tokyo, Japan). In the first step, the excitation and emission spectra of fluorophores, when in the lipid bilayer, were collected, and the wavelengths of maximum fluorescence intensities were determined. For NBD and Rh-B, the maximum fluorescence intensity was measured when the excitation/emission wavelength was equal to 467/531 and 569/589 nm, respectively. In experiments where the fluorescence resonance energy transfer between the two probes was evaluated, the excitation wavelength was set at 467 nm and emission spectra were acquired in the wavelength range from 520 to 630 nm.

## 3. Results and Discussion

### Quantitative Description of the Extrusion Process

**Figure 2:**
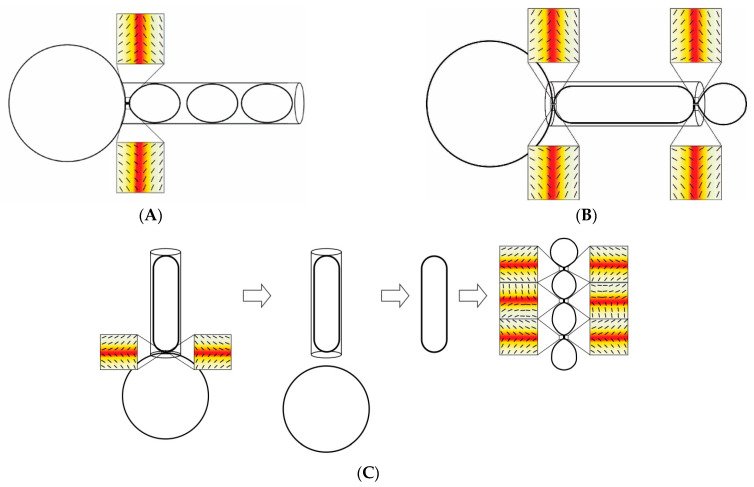
The schematic presentation of possible scenarios describing the liposome formation during the extrusion. Scheme (**A**)—unilamellar liposomes are formed at the pore opening and remain unaffected by subsequent steps. Scheme (**B**)—unilamellar liposomes are formed at the pore opening from the elongated lipid structure formed from fused liposomes. Scheme (**C**)—during the extrusion process, the elongated lipid structure is pulled from the multilamellar liposome and final unilamellar liposomes are a result of the budding process caused by the topological alterations inside the pore caused by defects in the lipid bilayer. The hypothetical orientational molecular ordering profiles in necklace-like buds/endovesicles show that topological anti-defects are accumulated in the necks. Consequently, the shape with prominent thin necks in scheme (**C**) may be transformed into distinct membranes as a result of the neck rupture. Positions of anti-defects are indicated by small squares. Orientational molecular ordering profiles with the superimposed nematic director fields in the vicinity of topological anti-defects are shown in magnifications. The color scale represents the degree of orientational order of the lipid bilayer. Dark red and white colors indicate the minimum (isotropy) and maximum (anisotropy) values of the orientational order, respectively. Figure was adapted from [81].

The extrusion process can be arbitrarily divided into the following stages: accumulation of lipid aggregates at the entrance of the pore (1), liposomes enter pores (2), passage of the vesicles through pores (3), and the release of liposomes on the other side of the extrusion membrane (4). Phase (1) can be neglected, as extrusion pores will be rapidly sealed by the multilamellar vesicles, as demonstrated by the dependence of the extrusion force on lipid concentration in the vesicle suspension [40]. The vesicle formation in the extrusion process depends on the intrinsic properties of lipids and the geometry of the extrusion system. Similar to the approach used in the GUV aspiration technique, the geometry of the liposome formation system can be reduced to the pore diameter [68,79]. The quantitative parameters characteristic for the lipid bilayer such as bending rigidity, rupture tension, and water permeability will also affect the final liposome topology [79,80]. Consequently, the average vesicle size will predominantly be affected by the extrusion pore diameter and the lipid composition. The dependence of liposome size on the lipid composition raises the issue of the mechanism of the liposome formation process. In this paper, the membrane cis and trans sides address surfaces where liposomes enter and leave the pore, respectively. Three scenarios can be proposed for the formation of liposomes (Figure 2).

1. The spheroidal liposome is formed at the pore (on the cis side) opening and remains intact through the remaining stages of the extrusion process.
2. The tubular liposome is formed at the pore opening (on the cis side) and the final vesicle is formed at the exit of the pore [63,82].
3. The tubular liposome is formed at the pore opening, but the stringed condition inside the pore forces the tubular structure to rearrange into unilamellar vesicles inside the pore [63].

All three scenarios account for the dependence of the final vesicle topology on the properties of the lipid bilayer and pore diameter. In the first scenario, the bending rigidity and the tensile strength of the lipid bilayer are factors affecting the final liposome size. The vesicle is formed when the resistance of the multilamellar vesicle at the surface of the extrusion membrane exceeds the tensile strength of the lipid bilayer. After that, the size of vesicles remains unchanged throughout the remaining stages of the extrusion process. This scenario has quantitative consequences for the recalibration process when the size of the unilamellar vesicle is altered due to the passage through the pore with smaller diameter. In this case, a unilamellar liposome breaks at the pore opening and a liposome with smaller size is formed. The remaining lipids can form liposomes on their own or fuse with other lipids present at the membrane surface. Each event will produce different experimental outcomes. The first possibility will produce two populations of liposomes and there would be no lipid mixing. In the second possibility, a single liposome population will form and lipids will mix in the process. In the second scenario, the elongated lipid structure forms at the pore opening. Liposomes are formed at the trans side of the membrane and the resulting unilamellar liposomes’ size will depend on the pore diameter mechanics of the lipid bilayer and thermal fluctuations of the liposome wall at the pore opening [63,81]. In this case, the sizes of liposomes will likely exceed the pore diameter. In this scenario, the recalibration will produce liposomes similar to those in the first scenario. In the third scenario, liposomes will form from the tubular structure inside the pore as a result of stringed conditions imposed by the pore wall generating spontaneous defects in the lipid bilayer, finally leading to vesicle formation. In this case, the size of the resulting liposomes will not exceed pore diameter.

Therefore, the determination of the liposome formation and recalibration mechanisms can be determined based on the populational analysis of produced vesicles and by measuring the stability of vesicles with respect to their lipid composition.

**Figure 3:**
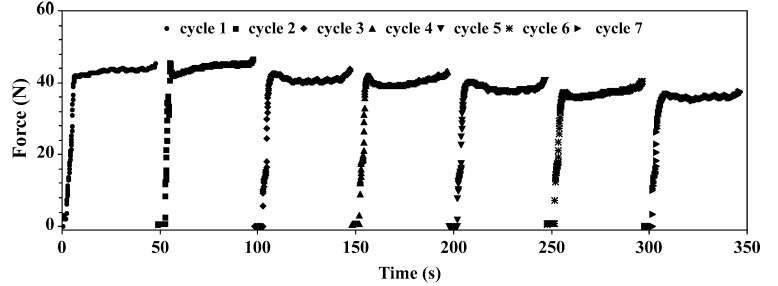
Example of raw data obtained during 7 cycles of the extrusion process of liposomes formed from POPC (1-palmitoyl-2-oleoyl-sn-glycero-3-phosphocholine). The measurements were performed at room temperature, with the volumetric flow and filter pore diameter equal to 0.48 mL/(min·cm^2^) and 0.08 µm, respectively.

Recently, the quantitative extrusion device has been introduced (Figure 1). The device is equipped with several features, which are useful in quantitative characterization of the liposome formation process, such as mechanics of a lipid bilayer. In recently presented studies, the molecular mechanism of the liposome formation process in the extrusion pores has been proposed along with the method of the bending rigidity determination [63]. The bending rigidity is a parameter used to quantitate lipid bilayer propensity for deformation. The main advantage of the method is that the measured values are averaged over a large population of vesicles, contrary to other approaches, where the bending rigidity is determined for a set of vesicles arbitrarily selected for the measurement from a large and frequently heterogeneous population [63]. The device is capable of measuring the force required to support a predetermined volumetric flow of liposome suspension through the extrusion membrane. In extrusion, the energy is dissipated due to the friction between liposomes and the pore wall, the bulk viscosity within the experimental systems, and rearrangements of lipid aggregates. The intrinsic energy dissipation of the device itself can be easily determined by the dedicated control experiment when no lipid is present. The energy-consuming liposome formation processes are: the deformation of lipid bilayers, pressure-driven water flow across a lipid bilayer, elongation, breaking the membrane, and flow vesicles through the pore. Finally, a vesicle leaving the pore recovers its specific topology, which depends mainly on temperature and membrane-intrinsic properties [80]. The typical dependence of extrusion force on time, when the volumetric flow is fixed, is presented in Figure 3. The extrusion force increases rapidly followed by a period when it stabilizes for the duration of the extrusion process.

In order to demonstrate the method reliability, the dependence of the extrusion force, needed to maintain the constant volumetric flow of liposome suspension, on the lipid composition was measured. For that purpose, the extensively studied and well-characterized cholesterol-phosphatidylcholine mixtures were selected [38,41,83,84].

**Figure 4:**
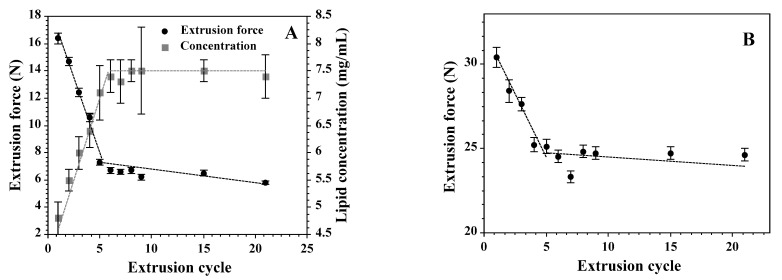

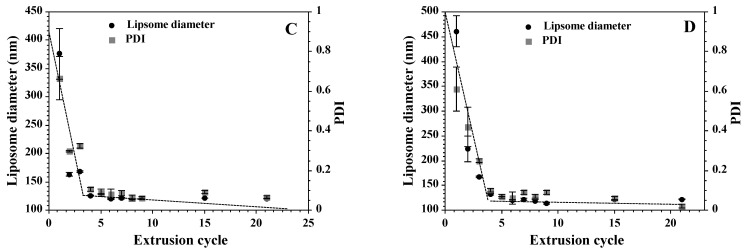
The dependence of the extrusion force on the cycle number when the liposomes are formed from POPC (**A**) or a mixture of POPC and cholesterol (70:30, mol:mol) (**B**). Panel A also shows the quantity of lipids crossing the membrane during each cycle. Panels (**C**,**D**) show the average size and PDI (Polydispersity Index) as evaluated using the dynamic light scattering method. The value of the extrusion force was corrected for the intrinsic resistance of the instrument. The measurements were performed at room temperature and the volumetric flow was equal to 0.48 mL/(min·cm^2^).

When designing the experimental protocol, we have taken advantage of the fact that the extrusion process consists of a series of cycles. Each cycle is performed upon a slightly different sample. The difference results from the growing fraction of unilamellar liposomes in the suspension. Figure 4A, in addition to the extrusion force, shows that the quantity of lipid on the trans side of the extrusion membrane increases when the number of extrusion cycles reaches saturation after approximately the sixth cycle. At the same time, the diameter of liposomes approaches the size of the membrane pores and the liposome population becomes homogeneous, as demonstrated by decreasing PDI values (Figure 4B). Similar observations have been reported by others [65,68,77,85]. The character of the dependence of liposome quality on the number of extrusion cycles is invariant with respect to the lipid composition used. Figure 4A,C show that the character of the dependence remains unchanged for liposomes formed from POPC or from the mixture of POPC and cholesterol. However, the force required to extrude vesicles is much higher when POPC/cholesterol is pushed through the membrane. The observed difference can be assigned to the difference in the lipid bilayer mechanics, in agreement with observations reported by others [78]. In addition, Figure 4 demonstrates that two qualitatively different vesicle suspensions can be defined: multilamellar vesicles in cycle one and unilamellar vesicles in cycle six and later. Consequently, the extrusion force in cycles 1 and 6 can be used to measure two different processes characterized by different properties of a lipid bilayer: the force required to push a multilamellar vesicle across the filter membrane will result in both rupture of the lipid bilayer, as required for the unilamellar vesicle formation, and lipid bilayer deformation upon entering the pore of the extrusion membrane. In the sixth cycle, on the other hand, only the lipid bilayer deformation is required. Consequently, the extrusion force in cycles 1 and 6 can be used to evaluate two different parameters, the tensile strength and bending rigidity, respectively. The cycle differentiation has been confirmed with an ANOVA test. For practical purposes, the tensile strength can be defined in terms of the membrane stretching resistance:

(2) $$E_{exp}=\frac{1}{2}K_{A}{\left(\frac{A-A_{0}}{A_{0}}\right)}^{2}$$

where the stretching elastic energy per unit area *E_exp_* depends on the change in the relative area (*A*_0_ and *A* are surface areas of the relaxed and stressed membrane, respectively) and material parameter (membrane compression modulus *K_A_*), whereas the bending deformation can be described in terms of Helfrich’s model, where the bilayers are characterized by two principal local curvatures *C_1_* and *C_2_* and three phenomenological parameters, the bending rigidity ($\kappa_{b}$), the Gaussian elastic modulus ($\kappa_{G}$), and the spontaneous curvature (*C_0_*) [86]:

(3) $$E_{bend}=\frac{1}{2}\kappa_{b}{\left(C_{1}+C_{2}-C_{0}\right)}^{2}+\kappa_{G}C_{1}C_{2}$$

As shown in Figure 4A, the average extrusion force for POPC vesicles monotonically decreases from the value of 16.4 N in the first cycle down to 6.7 N in the sixth cycle, showing that irreversible processes (liposome formation) contribute around 10 N to the force required to sustain the constant volumetric flow (Figure 4A). The remaining 6.7 N originates from the resistance of the device and reversible process (deformation of the lipid bilayer). Experimental results regarding topological alterations of the lipid bilayer driven by a hydrostatic pressure difference, as in the extrusion process, depend on the lipid bilayer-intrinsic properties such as mechanics, water permeability, as well as instrumental settings (temperature or volumetric flow) [79,87]. In light of the presented data, the irreversible process (liposome formation) will depend on lipid bilayer deformation upon entering the membrane pore and water fluxes, so adequate aqueous volumes would facilitate protrusion formation followed by the unilamellar vesicle budding from multilamellar structures. The relative contribution of these factors to the vesicle formation process [68,88] justify the assumption that the hydrostatic pressure difference needed for the constant flow of the liposome suspension across the extrusion membrane depends predominantly on the lipid bilayer tensile strength and bending rigidity. It has been assumed that the two processes are additive. The tensile strength can be described by the following equation [68]:

(4) $$∆P\approx 2\sigma_{m}\frac{1}{R_{p}}$$

where ∆*P* stands (approximately) for the hydrostatic pressure difference across the extrusion membrane, $R_{p}$ stands for the radius of the extrusion pore, and the effective lateral tension ($\sigma_{m}$) includes surface tensions from both surfaces and the contribution from the stretching of the membrane.

The hydrostatic pressure difference needed for the liposome formation can be extracted from the dependence of the extrusion force on time in the first cycle (Figure 3), where the hydrostatic pressure difference, after rapid increase, stabilizes for the duration of sample extrusion. The constant volumetric flow reflects the rate of unilamellar liposomes formation when lipid aggregates, before entering the extrusion membrane, are in the form of large multilamellar structures. In subsequent passages, the growing fraction of lipids form unilamellar vesicles, as reflected by the decreasing hydrostatic pressure difference needed to maintain the constant volumetric flow (Figure 4A). Interestingly, the dependence of the extrusion force on the number of passages through the membrane is composed of two phases, a rapid decrease in the extrusion force followed by a slower decline (Figure 4A,C). The first process correlates with the quantity of lipids on the “trans” side of the membrane (Figure 4A). The slower process can be assigned to the improvement of the liposome homogeneity.

To demonstrate that experimental data obtained using the extrusion method reflect the lipid bilayer mechanics, the well-characterized experimental models, namely liposomes formed from various mixtures of POPC and cholesterol, were used. It has been established by others that the addition of cholesterol changes significantly the lipid bilayer properties. Specifically, the bending rigidity and tensile strength are elevated, whereas the water permeability decreases [32,79,89,90].

**Figure 5:**
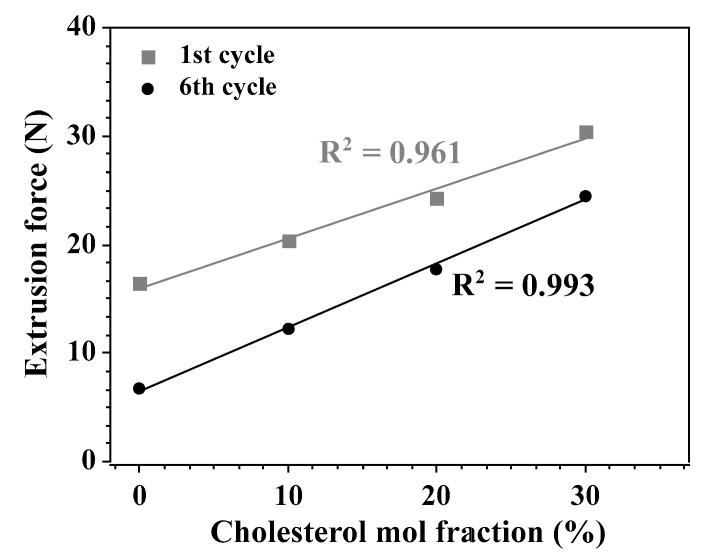
The dependence of the extrusion force in 1st and 6th cycles on the quantity of cholesterol in the lipid bilayer. The measurements were performed at room temperature and the volumetric flow was equal to 0.48 mL/(min·cm^2^). R indicates the coefficient of determination of the linear regression model.

**Figure 6:**
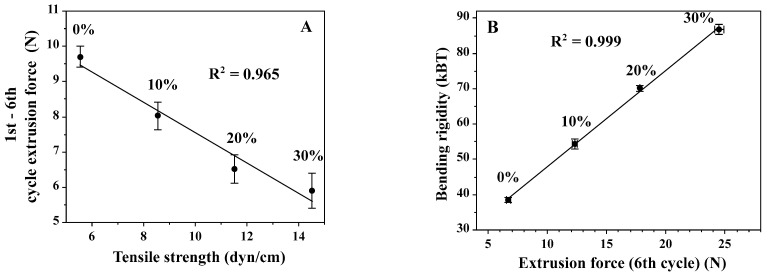
Correlations between extrusion forces and tensile strength (**A**) and bending rigidity (**B**). Panel A shows the correlation between tensile strength and extrusion force in the 1st cycle determined for the membrane with different contents of cholesterol [89]. The right panel shows the dependence of the bending rigidity on the cholesterol content in the membrane, as well as the correlation between the extrusion force measured and the tensile strength derived from the literature [32]. The measurements were performed at room temperature and the volumetric flow was equal to 0.48 mL/(min·cm^2^).

It was observed (Figure 5) that the dependence of extrusion forces during the first and sixth cycles on cholesterol membrane content are linear. This indicates that the bending rigidity and the tensile strength are both a linear function of the cholesterol content in the membrane. This qualitatively agrees with data presented by others [32,89]. Figure 6 shows the correlation between the extrusion forces of tensile strength and bending rigidity of membranes differing in the cholesterol content.

As stated before, the extrusion force in the first cycle reflects the resilience of the lipid bilayer to both deformation and rupture [89], whereas the extrusion force in the sixth cycle reflects mainly the pressure difference needed to deform vesicles and push them through the pore; therefore, it can be correlated with the bending rigidity coefficient (Figure 6). The dependence of the bending rigidity coefficient on the extrusion force in the sixth cycle for various membranes differing with respect to the cholesterol content is linear. The numerical values of the bending rigidity were borrowed from the literature [32]. The difference between extrusion forces in the first and sixth cycles is expected to correlate with the tensile strength. The correlation between values of the extrusion force and the tensile strength determined by others, for membranes differing with respect to the cholesterol content, is also linear (Figure 6A) [89]. However, in this case, when the cholesterol content in the membrane rises, the corrected extrusion force (the difference between cycles 6 and 1) decreases. This is an unexpected result as it has been observed, using GUV, as an experimental model, that the tensile strength increases with the cholesterol content [89]. It is well established that the presence of cholesterol in the lipid bilayer changes its properties, including decreased permeability, increased bending rigidity coefficient, and a change in the organization of hydrocarbon chains, as demonstrated by the modification of the main phase transition. The effect of cholesterol on membrane mechanics is complex and varies depending on the model used, lipid composition of the bilayer, and properties selected for analysis (for example, bending rigidity versus friction between monolayers) [81,89,90]. During extrusion, no single property but their combination is relevant, including the permeability to water, bending rigidity, and resistance to lateral and bending stresses [91]. The unexpected reduction in the tensile strength by rising the cholesterol content in the membrane, as measured with the extrusion method, can be rationalized by considering the water flux accompanying the liposome formation process. Cholesterol, by reducing the water flow across the lipid bilayer, changes the dynamics of the liposome formation process. The insufficient amount of water available for vesicle formation at the pore opening may result with the premature rupture of the membrane in the extrusion pore. The deficiency of the extrusion method with respect to the measure of the tensile strength requires further experimental clarification.

**Figure 7:**
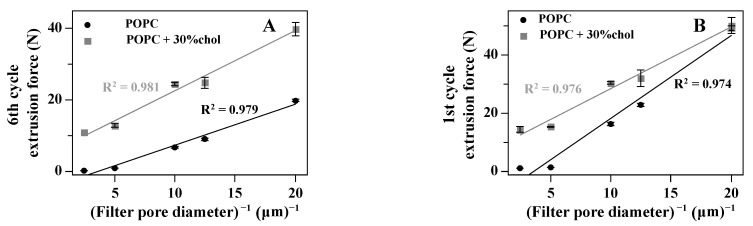

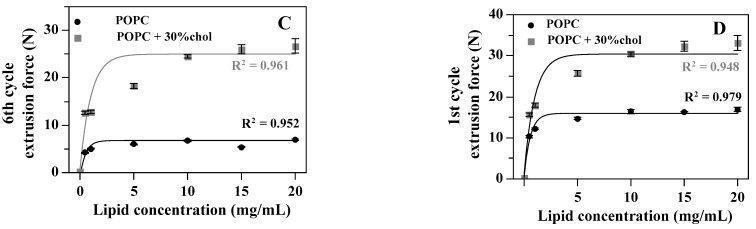
The effect of cholesterol on the dependence of the extrusion force on the extrusion pore size (sixth cycle (**A**) and firs cycle (**B**)) and lipid concentration (sixth cycle (**C**) and first cycle (**D**)).

When cholesterol is present in the lipid bilayer, the extrusion force is significantly higher when liposomes are extruded through the membrane with small pores. When membrane pores are small, the difference in values of the first-cycle extrusion force between the vesicle with and without cholesterol is negligible (Figure 7). When the dependence of the extrusion force on lipid concentration is evaluated, it can be seen that for POPC vesicles, the extrusion force is invariant on lipid concentration, whereas when cholesterol is present, the extrusion force increases monotonically in lipid concentration, showing that, in addition to the extrusion process, other processes occurring on the cis surface of the extrusion membrane may affect the measured extrusion force value. Comparing the dependence of the extrusion forces in the first and sixth cycles, it can be observed that for POPC vesicles, the extrusion force is higher for smaller pores and is smaller for the sixth cycle. Extrusion forces for POPC vesicles do not depend on the lipid concentration and their values are greater in the first cycle. When cholesterol is present, in the membrane, the extrusion force in all cases is higher than that for POPC alone. However, for small membrane pores, the extrusion force in the first cycle is similar to that when the vesicle does not contain cholesterol. No such effect is observed when the extrusion force during the sixth cycle is compared (Figure 7). Again, this result can be rationalized by the alteration of the lipid bilayer permeability to water by the presence of cholesterol, which will change the liposome formation dynamics.

## 4. Lipid Mixing in the Extrusion Process

As described earlier, the formation of unilamellar vesicles in the extrusion process requires massive molecular rearrangements [63,72,91,92]. There are two points in the experimental system where final vesicles may form: at the entry (cis side) and at the exit (trans side) of the pore. It has been postulated before that lipid molecules originating from MLVs on the cis side of the membrane are forced into the cylindrical pore of the extrusion membrane. The pore dimensions are: 0.1 and about 10 μm, for pore diameter and length, respectively. During extrusion, elongated lipid structures or final liposomes flow through the pore powered by the hydrostatic pressure difference. At the pore exit, the elongated vesicle breaks down into smaller, unilamellar spherical liposomes with a diameter corresponding to the pore size [82]. The possible topological alterations, which may occur during the extrusion, are schematically presented in Figure 2.

The molecular mechanism of topological alterations upon imposed or released external forces has been correlated with the occurrence of local defects induced by the altered topology of the lipid bilayer [81].

Assuming that molecular processes during liposome recalibration do not differ significantly from those when unilamellar liposomes are formed from multilamellar vesicles, one can predict the outcome of the recalibration process. When a uniform population of unilamellar liposomes is forced through the extrusion membrane with pores of smaller sizes, it is expected that the liposome population is altered with respect to liposome size distribution. In addition, the exchange of lipids between vesicles may take place. Each scenario of the extrusion process will result in different outcomes regarding liposome size distribution and/or lipid mixing. When vesicles are formed from elongated lipid structures at the exit of the extrusion pore (Figure 2B), the resulting liposome population is expected to be homogenous, and there should be an extensive lipid mixing. When liposomes are formed at the entrance of the pore and there is no vesicle fusion at any point (Figure 2A), the outcome should be different. Liposomes will be recalibrated at the pore entrance and will pass the pore in the final form. As a result, there will be no lipid mixing between vesicles, and the resulting liposome suspension should consist of two liposomes populations: one that will correspond to the pore diameter of the membrane used for the recalibration, and the other population formed from the “left-over” lipids.

**Figure 8:**
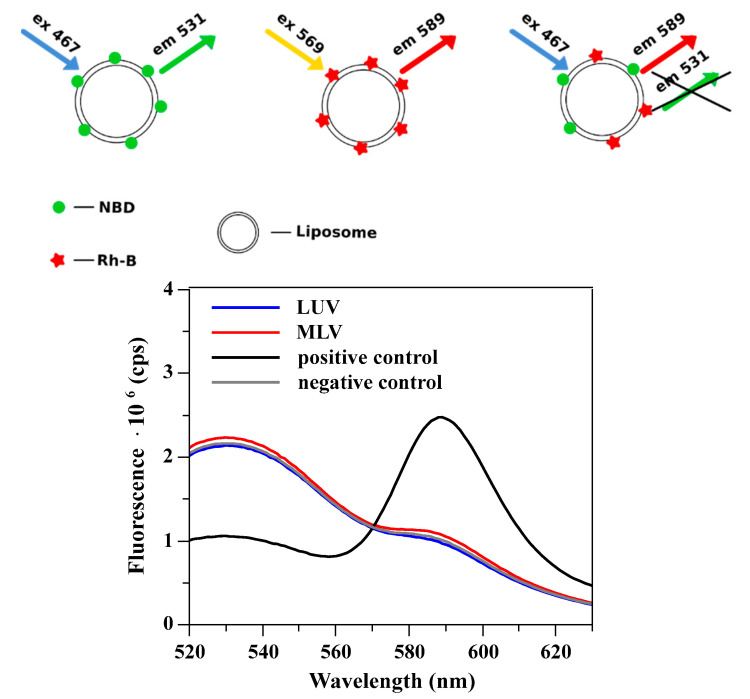
The upper panel shows the liposome labeling method. Liposomes were labeled with a single fluorescent dye or with both of them. Each liposome population has a different fluorescence fingerprint (lower panel). When liposomes were labeled with both dyes, an extensive energy transfer was observed (positive control). For comparison, the fluorescence of liposomes labeled with a single dye (large unilamellar vesicles (LUVs) labeled with either NBD (1-acyl-2-(6-[(7-nitro-2-1,3-benzoxadiazol-4-yl)amino]hexanoyl)-sn-glycero-3-phosphocholine) or Rh-B (18:1 Liss Rhod PE 1,2-dioleoyl-sn-glycero-3-phosphoethanolamine-N-(lissamine rhodamine B sulfonyl) ammonium salt) fluorescent probe) was mixed and the resulting emission spectrum is presented (negative control-labeled vesicles are mixed without fusing). When LUVs or MLVs (multilamellar vesicles) labeled with a single fluorescent dye were mixed and extruded together, the emission spectrum was similar to that of the negative control. The spontaneous exchange of labeled lipids or other membrane components was excluded due to their high logP (octanol-water partition coefficient) values [95,96].

**Figure 9:**
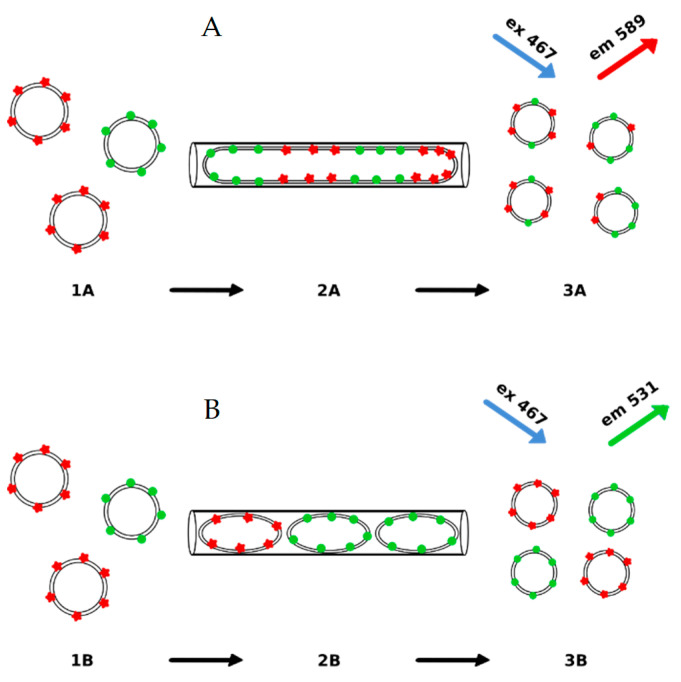
The effect of the liposome formation mechanism on the outcome of the fluorescence experiment when two populations of liposomes, each labeled with different fluorescence dyes, are mixed and extruded. (**A**) presents the scenario when e longated lipid structure (**2A**) forms from large or multilamellar liposomes (**1A**). When leaving the pore liposomes bud at the pore exit from the elongated lipid aggregate (**3A**). In this case a significant energy transfer is expected. In the second scenario (**B**). LUV liposomes are formed at the pore entrance from multilamellar or large liposomes (**1B**), remain unchanged when passing (**2B**) and finally leaving (**3B**) the pore. In this case, the extrusion of the differently labeled liposomes would produce vesicles without lipid mixing. Therefore, no energy transfer between the two labels is expected.

Lipid mixing and uniformity of the liposome population can be experimentally tested. The possible lipid mixing upon formation of the elongated lipid structure inside the membrane pore can be quantitated using the well-established fluorescence method [93]. In short, vesicles are decorated with two fluorescently labeled lipids. The fluorescence dyes (NBD and Rhodamine) are a well-established combination for detecting the lipid mixing [94]. In order to perform the experiment, three populations of vesicles were prepared, as schematically presented in Figure 8. The method is described in detail in Supplementary Materials. The fluorescence resonance energy transfer (FRET) was used as a quantitative measure of the extent of lipid mixing. In the experiment, two populations of multilamellar or unilamellar liposomes labeled with NBD or Rhodamine were extruded together, and the resulting fluorescence was measured. The outcome of the experiment, as predicted by the liposome formation mechanisms in its final stage, is schematically presented in Figure 9. The vesicles fusion-driven formation of the elongated lipid structure inside the pore should result in lipid mixing, as illustrated in Figure 9A. When the sample is exposed to the excitation light with the wavelength specific to NBD dye (467 nm), the emission spectrum should reflect the energy transfer to rhodamine dye, as shown in Figure 8. When liposomes are formed at the entrance of the pore, the expected outcome of the fluorescent experiment is schematically shown in Figure 9B. The two possible mechanisms of the liposome formation process, when two populations of vesicles labeled with different dyes are extruded together after mixing, will result in different outcomes (Figure 8 and Figure 9).

**Figure 10:**
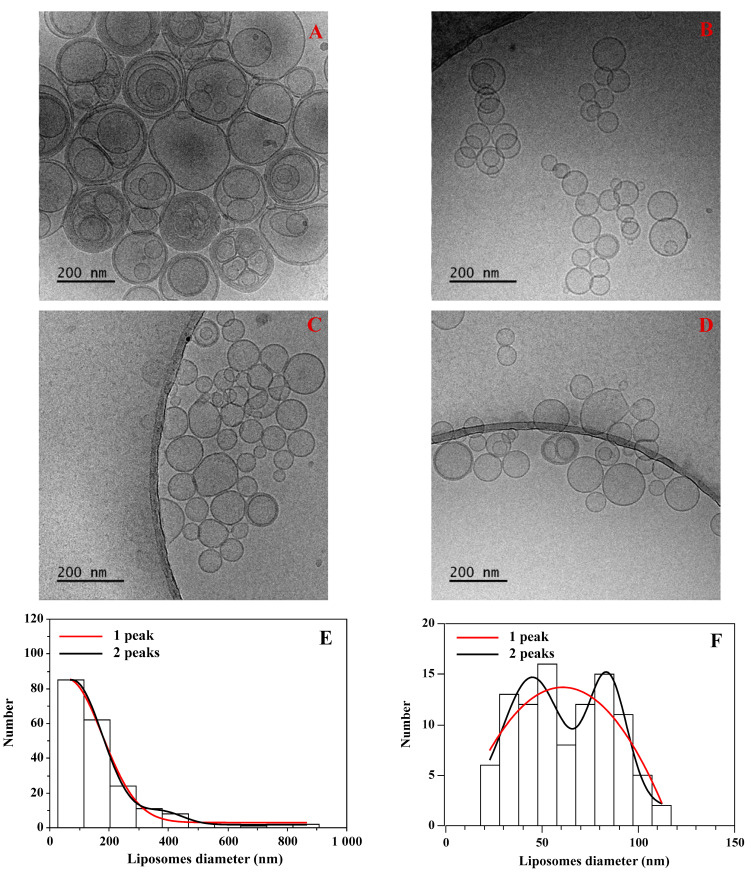
Examples of TEM (Transmission electron microscopy) images (Tecnai F20 X TWIN microscope, FEI Company, Hillsboro, OR, USA) collected for two liposome samples: (**A**) homogenously extruded vesicles (200 nm pores), followed by images when liposomes were recalibrated through 50 nm (**B**), 80 nm (**C**) and 100 nm (**D**) membranes. Lower panels show examples of histograms constructed from TEM images for liposomes extruded through 200 nm pores (**E**) followed by extrusion through 50 nm pores (**F**). Histograms were fitted with 1 or 2 Gaussian distributions.

The experiment has shown that when a mixture of two differently labeled populations of multilamellar liposomes was extruded, no energy transfer between the two dyes was detected (Figures S1–S4, Table S1, Supplementary Material). This indicates that unilamellar liposomes formed during the extrusion process originate from a single multilamellar structure, and no lipid mixing is taking place. A similar result was obtained when two populations of 130 nm unilamellar liposomes were pushed through the extrusion membrane with smaller pores (50 or 80 nm). Here, again, no detectable lipid exchange was measured. The fluorescence experiment points to the scenario when liposomes are formed at the pore entrance, and there is no lipid mixing during the recalibration process. Results of the fluorescence experiments indicate that in the recalibration process, there are two liposome populations differing with respect to the average sizes. In order to test the prediction, quantitative analysis of the electron microcopy images and dynamic light scattering measurements were performed. Figure 10 and Table 1 show results of the examination of the liposome population when unilamellar liposomes with well-defined sizes (137 or 187 nm) are pushed through membranes with similar or smaller pores (50 or 80 nm).

Table 1 shows that the average liposome size determined with the two experimental techniques agrees only when the liposome suspension was recalibrated through a membrane with pore sizes equal to those used during the extrusion process (100 or 200 nm). Interestingly, there are large discrepancies between average sizes determined using DLS and TEM techniques. These results show that the scenario when liposomes are formed at the pore opening is most likely. According to the scenario, homogenous populations of liposomes recalibrated through the membrane with smaller pore sizes become heterogenous, as demonstrated by cryo-TEM images. In the cryo-TEM technique, the liposome size distribution was derived from obtained images. Next, the size distribution was fitted with the single or two population models, and the quality of fitting was used as a method for the differentiation between a homogeneous and heterogeneous vesicle population. The liposome population was quantitatively evaluated using the quality of fitting with a single or two Gaussian distributions. The result of the analysis is presented in Table 2.

Analysis of the TEM images obtained for vesicles produced using the extrusion and recalibration processes showed that liposome population is homogeneous, when produced using a single extrusion procedure. When the preformed homogenous unilamellar vesicle population is recalibrated using the membrane with smaller pores, the liposome population becomes heterogeneous, as demonstrated in Figure 10 and Table 2. The effect is especially pronounced when liposome suspensions extruded through 100 or 200 nm pores are recalibrated through a 50 nm filter.

## 5. Conclusions

The extrusion process, despite its application for liposome formation for decades, is still not well understood. In the paper, a new experimental method for the quantification of the extrusion process was introduced and its effectiveness and reliability demonstrated. The method is based on the measurement of the extrusion forces necessary to ensure the constant volumetric flow through the extrusion filter. The device made it possible to develop the experimental procedure for the quantitative evaluation of mechanical properties of a lipid bilayer, a task difficult to achieve via other means. The linear correlation between bending rigidity, tensile strength, and extrusion forces was demonstrated. Next, the method was used to elaborate on the molecular mechanisms leading to the vesicle formation during the extrusion process. Using the fluorescence energy transfer method, it was demonstrated that there is no liposome fusion and/or lipid mixing inside the extrusion pore, indicating that liposomes following formation remain unchanged even when exposed to mechanical stress. The observation shows that the liposome recalibration process is a result of their fractionation resulting from the degraded homogeneity of the liposome suspension. These findings are relevant for the development and/or application of the liposome formation process using the extrusion method, both for basic science, as a means to produce experimental models, or for applied sciences, where liposomes are intended for pharmacological applications.

## Figures and Tables

**Table 1:** The liposomes size determined by two techniques: DLS (Dynamic Light Scattering) and cryo-TEM (Cryogenic electron microscopy). Polydispersity index (PDI) was also shown in the table.

Liposome Suspension	Average Size Determined by DLS Technique (nm) [97] (PDI)	Average Size Determined by cryo-TEM Technique (nm) [97]
LUV100 (liposomes prepared by extrusion through a 100 nm pore/filter)	137 (0.11)	133
LUV200 (liposomes prepared by extrusion through a 200 nm pore/filter)	187 (0.15)	176
R100-50 (extrusion of LUV100 liposomes through a 50 nm pore/filter)	88 (0.05)	62
R100-80 (extrusion of LUV100 liposomes through a 80 nm pore/filter)	118 (0.07)	74
R200-50 (extrusion of LUV200 liposomes through a 50 nm pore/filter)	88 (0.04)	63
R200-80 (extrusion of LUV200 liposomes through a 80 nm pore/filter)	116 (0.06)	80
R200-100 (extrusion of LUV200 liposomes through a 100 nm pore/filter)	132 (0.11)	86

**Table 2:** The correlation coefficient (R^2^) for the approximation of the liposome size distribution with one or two Gaussian distributions.

Probe	Single-Gaussian Fitting	Two-Gaussian Fitting
LUV100	0.999	0.995
LUV200	0.998	0.989
R100-50	0.336	0.891
R100-80	0.951	0.973
R200-50	0.377	0.884
R200-80	0.813	0.955
R200-100	0.891	0.962

## Data Availability

Data is contained within the article or Supplementary Material.

## Supplementary Materials

The following are available online at https://www.mdpi.com/article/10.3390/ma14154278/s1, Figure S1. (A)–The fluorescence emission spectra of liposomes labeled with NBD or Rh-B when excited at 467 or 569 nm, respectively. (B)–The fluorescence emission spectra of liposomes labeled with NBD or Rh-B or with both probes when excited at 467 nm, Figure S2. Panel (A)—Liposomes labelled with NBD or Rh-B were mixed and the fluorescence emission spectra were measured (excitation wavelength equals to 467 nm) after 1 and 72 h. In either case no energy transfer was detected. The mix of liposomes labeled with different fluorescent probes where no energy transfer was detected was used as the negative control. Panel (B)–200 nm LUVs labeled with the NBD or Rh-B probe were prepared. Then the samples were mixed and extruded through filters with a pore diameter of 100, 80 or 50 nm, Figure S3. (A)—The fluorescence emission spectra of sample containing from 0 to 100% liposomes with intrinsic FRET effect. (B)—Effect of number of liposomes with the intrinsic FRET effect on the ratio of fluorescence intensities at 589 and 531 nm when excited at 467 nm, Figure S4. (A)—The fluorescence emission spectra of samples containing liposomes with the Rh-B which concentration equals from 0 to 0.5 mol% (each sample contains 0.5 mol% NBD). (B)—The effect of the Rh-B concentration on the ration of fluorescence intensities at 589 and 531 nm, when excited at 467 nm, Table S1. The quantitative analysis of FRET was determined by the the ratio of the fluorescence intensity at 589 nm (Rh-B emission) to 531 nm (NBD emission) after excitation at 467 nm.
