# Supplementary material for: The Elucidation of the Molecular Mechanism of the Extrusion Process
Source: Materials (Basel). 2021 Jul 30;14(15):4278. doi: 10.3390/ma14154278 (PMC8348501; doi:10.3390/ma14154278)
Supplement: Supplementary file 1 [file materials-14-04278-s001.zip › materials-1276775-supplementary.pdf]

materials

MDPI

Supplementary Material

The Elucidation of the Molecular Mechanism of the Extrusion Process

Joanna Doskocz [1,2,*], Paulina Dałek [1,2], Magdalena Przybyło [1,2], Barbara Trzebicka [3], Aleksander Foryś [3], Anastasiia Kobyliukh [3], Aleš Iglič [4,5] and Marek Langner [1,2]

[1] Department of Biomedical Engineering, Faculty of Fundamental Problems of Technology Wrocław, University of Science and Technology, pl. Grunwaldzki 13, 50-377 Wrocław, Poland; paulina.dalek@pwr.edu.pl (P.D.); magdalena.przybylo@pwr.edu.pl (M.P.); marek.langner@pwr.edu.pl (M.L.)
[2] Lipid Systems sp. z o.o., ul. Krzemieniecka 48C, 54-613 Wrocław, Poland
[3] Centre of Polymers and Carbon Materials, Polish Academy of Sciences, ul. M. Curie-Sklodowskiej, 41-819 Zabrze, Poland; btrzebicka@cmpw-pan.edu.pl (B.T.); aforys@cmpw-pan.edu.pl (A.F.); akobyliukh@cmpw-pan.edu.pl (A.K.)
[4] Laboratory of Physics, Department of Fundamentals of Electrical Engineering, Mathematics and Physics, Faculty of Electrical Engineering, University of Ljubljana, Tržaška 25, SI-1000 Ljubljana, Slovenia; ales.iglic@fe.uni-lj.si
[5] Laboratory of Clinical Biophysics, Department of Orthopedics, Faculty of Medicine, University of Ljubljana, Zaloška 9, SI-1000 Ljubljana, Slovenia
* Correspondence: joanna.doskocz@pwr.edu.pl

The mechanism of liposome extrusion process based on the fusion of liposomes and formation of elongated lipid structures inside the membrane pore during the extrusion process were verified using the lipid mixing essay. Specifically, two populations of liposomes containing different fluorescently labelled lipids were extruded together. The extend of resonance energy transfer (FRET) was used as a quantitative measure of the lipid mixing. For that purpose two fluorophores has been used; NBD and Rh-B probes.

The fluorescence of LUV labelled by NBD or Rh-B is shown in Figure S1A. For NBD and Rh-B the maximum fluorescence intensity was measured when excitation/emission wavelength equals 467/531 and 569/589 nm, respectively. The fluorescence emission when liposomes were labelled with both probes is shown in Figure S1B. The obtained emission spectra show the efficient FRET effect between NBD and Rh-B probes. Liposomes labelled with both fluorescent probes will be considered as the positive control.

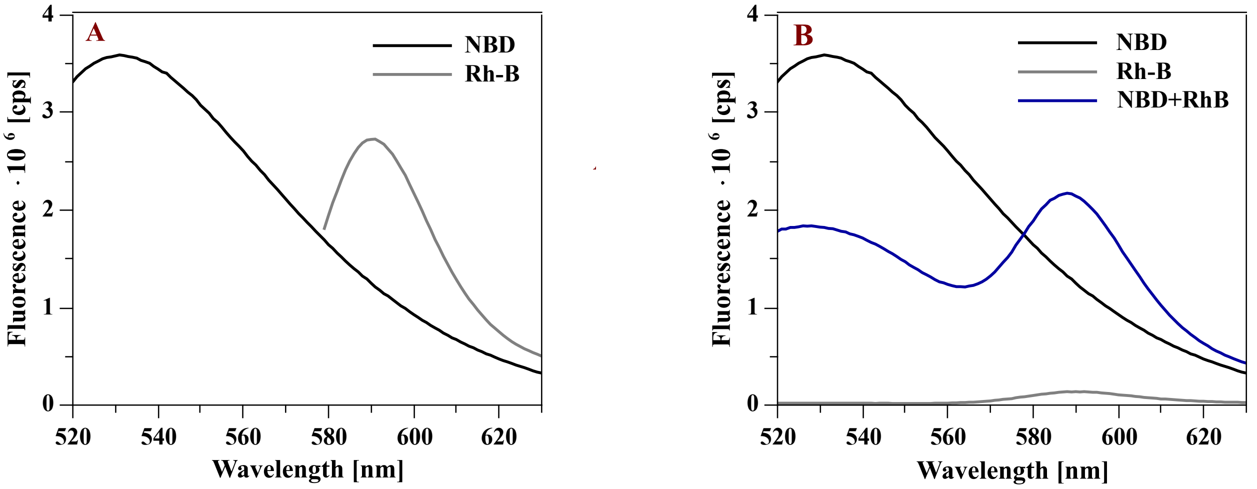

Figure S1. (**A**)–The fluorescence emission spectra of liposomes labeled with NBD or Rh-B when excited at 467 or 569 nm, respectively. (**B**)–The fluorescence emission spectra of liposomes labeled with NBD or Rh-B or with both probes when excited at 467 nm.

The negative control, when no lipid mixing take place, consists of mixture of liposomes labeled with a single fluorescence probe. In this case no energy transfer was detected as shown in Figure S2B. The result did not change after incubation of liposome mixture for 72 h indicating that there are no spontaneous liposome fusion.

When mixture of LUVs or MLVs labelled with NBD or Rh-B probes were recalibrated or extruded there were no detectable energy transfer (Figure 8). The collected spectra were similar to that obtained for the negative control. The effect of the filter pore diameter on the efficiency of FRET was also checked. For this purpose, LUVs labelled by the NBD or Rh-B probe were prepared using membrane with 200 nm pores. Then the samples were mixed and extruded through filters with a pore diameter of 100, 80 or 50 nm. The obtained results are shown in Figure 2SB. The quantitative analysis of the FRET effect was quantitated using the ratio of the fluorescence intensity at 589 nm (Rh-B emission) to 531 nm (NBD emission) after excitation with 467 nm. The obtained result is shown in Table S1.

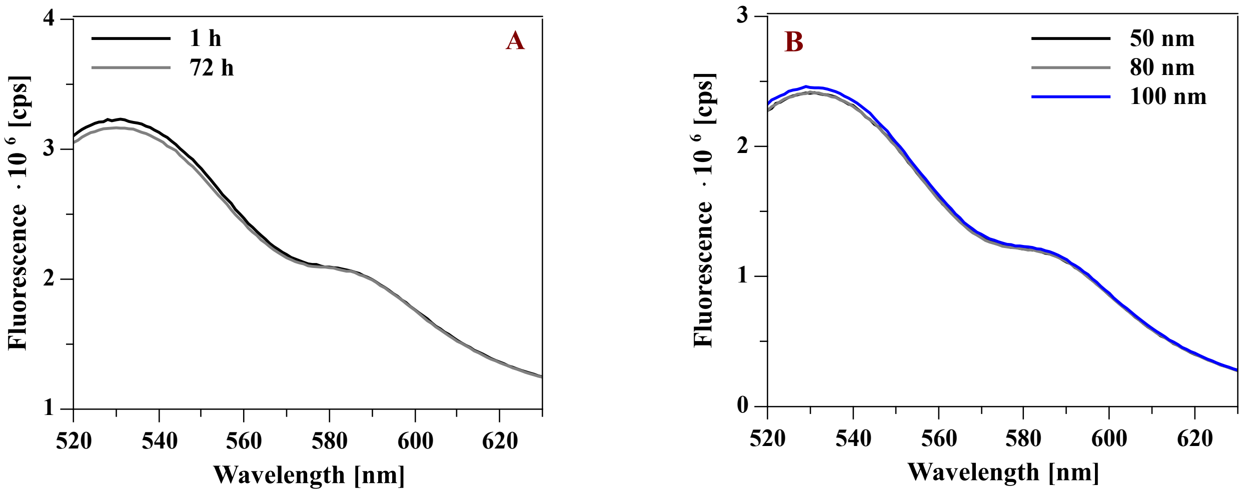

Figure S2. Panel (**A**)—Liposomes labelled with NBD or Rh-B were mixed and the fluorescence emission spectra were measured (excitation wavelength equals to 467 nm) after 1 and 72 h. In either case no energy transfer was detected. The mix of liposomes labeled with different fluorescent probes where no energy transfer was detected was used as the negative control. Panel (**B**)—200 nm LUVs labeled with the NBD or Rh-B probe were prepared. Then the samples were mixed and extruded through filters with a pore diameter of 100, 80 or 50 nm.

Table S1. The quantitative analysis of FRET was determined by the the ratio of the fluorescence intensity at 589 nm (Rh-B emission) to 531 nm (NBD emission) after excitation at 467 nm.

**Probes**		**F_{589}/F_{531} (-)**
Positive control (liposomes labeled with two probes, Figure S1B)	-	1.10 ± 0.14
Negative control (mixed liposomes labeled with one of two probes, Figure S2A)	After 1 h	0.45 ± 0.03
	After 72 h	0.47 ± 0.03
Mixed liposomes labeled with different probes and then extruded (Figure 8)	LUV	0.45 ± 0.02
	MLV	0.48 ± 0.03
Mixed liposomes labeled with one probe and then extruded using filter pore with different size (Figure S2B)	100 nm	0.47 ± 0.01
	80 nm	0.47 ± 0.02
	50 nm	0.47 ± 0.02

In order to determine the detection limit of the FRET-based method (the minimum level of lipid mixing detectable with the FRET effect). For this purpose, liposomes labelled with NBD and Rh-B (the positive control) were mixed with Rh- or NBD-labeled liposomes (not FRET probes—negative control). As a result, samples containing from 0 to 100% liposomes with the maximum FRET effect were obtained. Then the fluorescence emission spectra were measured (Figure S3A) and the ratio of fluorescence intensities at 589 and 531 nm was determined (Figure S3B). The dependence of the ratio determined for liposome mixtures on the fraction of liposomes with intrinsic FRET effect is a linear function.

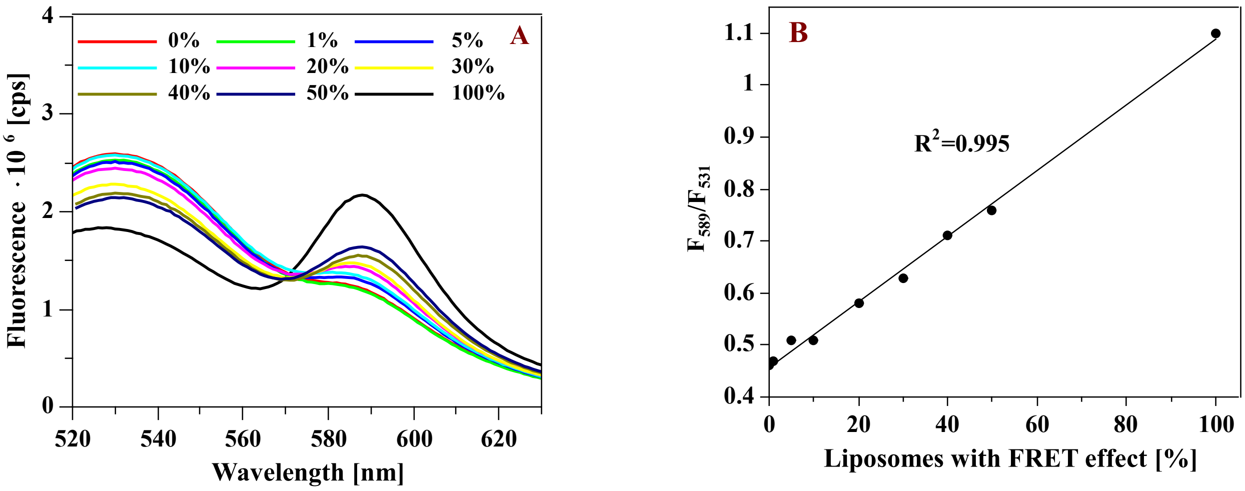

Figure S3. (**A**)—The fluorescence emission spectra of sample containing from 0 to 100% liposomes with intrinsic FRET effect. (**B**)—Effect of number of liposomes with the intrinsic FRET effect on the ratio of fluorescence intensities at 589 and 531 nm when excited at 467 nm.

Additionally, the minimum amount of Rh-B probe on the liposomes to observe the FRET effect was determined. For that purpose, liposomes labeled with NBD (constant value) and Rh-B (variable value) were prepared. Each sample contains the same number of liposomes. The NBD concentration equals 0.5 mol% and Rh-B concentration equals between 0 to 0.5 mol%. Then the sample fluorescence emission spectra were measured (Figure S4A) and the ratio of fluorescence intensities at 589 and 531 nm, when excited at 467 nm was determined (Figure S4B). Obtained data show that the dependence of the ration of the fraction of the acceptor (Rh-B) in the lipid bilayer can be approximated with the linear function.

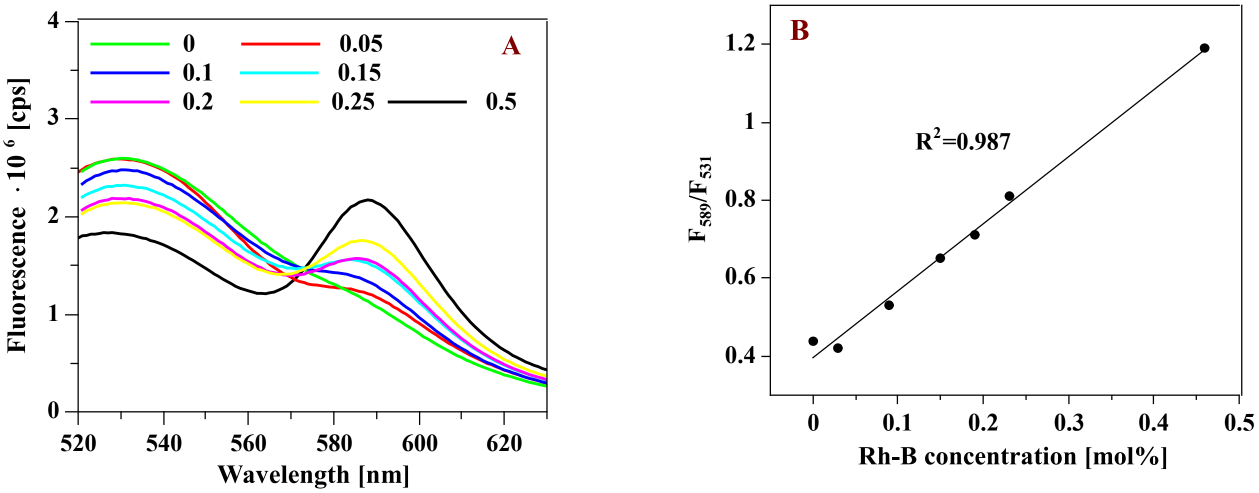

Figure S4. (**A**)—The fluorescence emission spectra of samples containing liposomes with the Rh-B which concentration equals from 0 to 0.5 mol% (each sample contains 0.5 mol% NBD). (**B**)—The effect of the Rh-B concentration on the ration of fluorescence intensities at 589 and 531 nm, when excited at 467 nm.

The method detection limit (*LOD*) was calculated based on equation:

$$LOD = \frac{3 \times sd}{a} \tag{1}$$

where *sd* and *a* indicate a standard deviation and the slope of the calibration curve, respectively.

Received results indicate that FRET effect is recorded if the sample contains at least 2.8% of liposomes labelled with Rh-B and NBD together. Additionally, the detection of FRET effect requires at least 0.06 mol% Rh-B in liposomes.
